# Editorial: Frontiers in diagnostic and therapeutic approaches in diabetic sensorimotor neuropathy

**DOI:** 10.3389/fendo.2023.1228101

**Published:** 2023-06-15

**Authors:** Péter Kempler, Adrienn Menyhárt, Viktor Horváth, Áron Tamás Kiss, Anna Erzsébet Körei

**Affiliations:** Department of Medicine and Oncology, Semmelweis University, Budapest, Hungary

**Keywords:** diabetic neuropathy, diabetic sensory neuropathy, metformin, B12 deficiency, meta-analysis, adenosine deaminase

Diabetic neuropathy is a classical interdisciplinary complication in the broadest sense. It still resides on the unattended borderline between internal medicine, including gastroenterology and neurology, as well as many other disciplines. Notwithstanding this, nerve damage is often described as the “forgotten” or “neglected complication” of diabetes (1).

Pain and unsteadiness are mainly responsible for impaired quality of life due to neuropathy, while neuropathic damage/deficit is primarily associated with poor prognosis. Among different types of nerve damage, cardiovascular autonomic neuropathy has been shown first to be related to poor prognosis and an increased risk of sudden death. In a 12-year-long prospective cohort study among type 1 diabetic patients, sensory damage was associated with a 1,72x higher mortality compared to patients with normal sensory function (2). Sensory neural damage has also been shown as an independent risk factor for myocardial infarction (3). Interestingly, the association between the frequency of myocardial infarction and symptoms of neuropathy was linear, while myocardial infarction frequency was exponentially related to the severity of neuropathic damage (3). According to results of a population-based cohort of patients with type 2 diabetes from the UK Clinical Practice Research Datalink (n = 49027, median follow-up: 5,5 years), the cumulative burden of microvascular disease including peripheral neuropathy significantly increased the risk of future cardiovascular events (4).

More recently, over a mean 9-year follow-up, we described a similarly strong association between DPN and all-cause mortality in patients with type 1 and type 2 diabetes mellitus (5). The results were adjusted for several factors including age, sex, anthropometric factors (height, BMI), lifestyle habits (current smoking and alcohol consumption), duration of diabetes, systolic and diastolic blood pressure, antihypertensive treatment, as well as comorbidities (measured using the simplified Charlson comorbidity index) and medications (lipid-lowering drugs, antianginal medications, antiarrhythmic drugs, platelet aggregation inhibitors, and anticoagulants). As part of the current Research Topic, our meta-analysis (Vági et al.) assessed the relationship between sensory neuropathy and mortality. Diabetes patients with DPN had an almost twofold higher mortality rate compared to those without DPN. This relationship was only partly explained by the baseline risk factors. The association was stronger in type 1 compared to type 2 diabetes. Until now, diagnostic laboratory methods have not yet been used in the diagnosis of DPN. Recently, serum neurofilament light chain has been described as a novel biomarker for early diabetic sensorimotor polyneuropathy (6). Higher levels of adenosine deaminase have been shown to be involved in metabolic and immunological abnormalities, which may contribute to inflammation (7, 8). Moreover, chronic subclinical inflammation plays a role in the pathogenesis of type 2 diabetes mellitus and in the development of its micro- and macrovascular complications. According to the results of Yu et al, increased levels of serum adenosine deaminase is associated with an increased risk of DPN in type 2 diabetes mellitus.

A new potential indicator of DPN has been described in another cross-sectional study: the findings of Yan et al suggest that the aspartate aminotransferase/alanine aminotransferase ratio (AAR) was associated with the presence of DPN. These results are significant from two perspectives. On the one hand, it is well known that AAR correlates with oxidative stress, chronic systemic inflammation, as well as a wide range of cardiometabolic consequences of insulin resistance, including non-alcoholic fatty liver disease. A connection between AAR and DPN has not been previously assessed, most likely as the clinical interest towards cardiovascular complications is currently greater compared to microvascular complications. On the other hand, both enzymes serve as the most commonly used liver enzymes reflecting hepatocellular injury, they are measured routinely among type 2 diabetic patients, so their assessment does not require additional costs.

Metformin is suggested to be used as the first oral glucose lowering drug worldwide. We disagree with the opinion that any other drug should be prioritized over metformin, unless it is not tolerated or its use is contraindicated. It has been shown that a 10-year survival rate was related to the glycaemic efficacy of the first 6 month of metformin treatment (9). The contraindications of metformin treatment in the past – advanced age, impaired renal function – have become much milder nowadays, while lactic acidosis is rarely seen. However, the importance of neuropathy, associated with metformin induced B12 deficiency has been underestimated until now. Although a clear relationship between metformin use and a higher risk of DPN was confirmed in a retrospective cohort study (10) involving veterans (n = 210.004) and this association was proven by other authors as well, most of the data in this respect are contradictory (11). Thus the paper of Yang et al deserves particular attention. Patients receiving metformin treatment had an 84% higher risk of DPN compared to those not using metformin. Moreover, the daily dose of metformin was positively associated with the risk of DPN. As one of the most relevant findings, patients of the metformin group taking vitamin B12 already at baseline had no increased risk of DPN. As a clear take home message of the study, diabetic patients receiving metformin treatment should take vitamin B12 as well.

The importance of lifestyle intervention is usually emphasized as an essential part of diabetes management. It is worth noting that the role of lifestyle intervention is not emphasized as much in the therapy of microvascular complications, despite its inclusion in a recently published international guideline on the diagnosis and treatment of DPN (12). Accordingly, the paper of Kender et al assessing the effect of periodic fasting on somatosensory nerve function is highly relevant. The results indicate that neither periodic fasting nor Mediterranean diet in the control group had any detrimental effects on somatosensory nerve function in the type 2 diabetic patients. These data are of importance from another aspect. Studies with apparently negative results are rarely published, despite their importance.

The clinical interest towards diabetic neuropathy has considerably increased due to the availability of some more or less effective treatments for managing this complication. As all relevant symptomatic agents are now already generic, there is an increased attention towards pathogenetic-oriented therapies. It should be noted that the so-called cardiovascular risk factors are global, micro- and macrovascular risk factors. With that in mind, blood pressure, lipids and smoking should be considered risk factors for the development and progression of DPN as well. As a special pathogenetic treatment, the antioxidant alpha-lipoic acid and the transketolase-activator benfotiamine should be mentioned, the latter inhibiting the harmful alternative pathways, such as the polyol-, hexosamine, protein kinase C and advanced glycation end product pathways. Tables and algorithms summarizing different treatments for DPN are usually too complicated and, as a consequence, difficult to use in everyday clinical practice. Therefore we suggest a simplified approach for the therapy of DPN (**Figure 1**).

**Figure 1:**
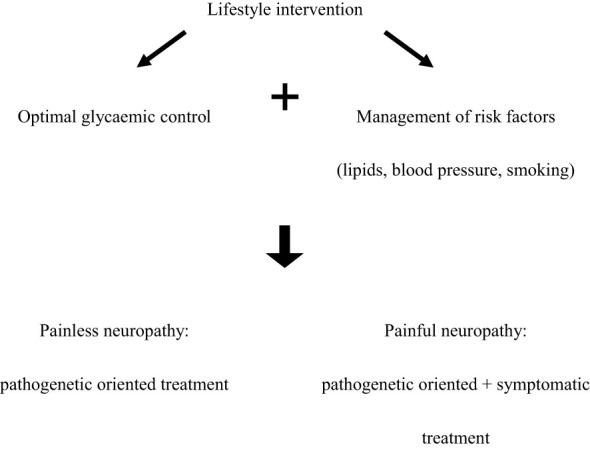
Simplified treatment algorithm for DPN.

## Author contributions

PK, AM, VH, ÁK and AE contributed to the conception and design of the editorial, and prepared the summaries of the papers of the Research Topic. The final manuscript has been prepared by PK and AE. All authors contributed to manuscript revision, read and approved the submitted version.
